# Synthesis and Characterization of Graphene Oxide Derivatives via Functionalization Reaction with Hexamethylene Diisocyanate

**DOI:** 10.3390/nano8110870

**Published:** 2018-10-23

**Authors:** Jose Antonio Luceño-Sánchez, Georgiana Maties, Camino Gonzalez-Arellano, Ana Maria Diez-Pascual

**Affiliations:** 1 Departamento de Química Analítica, Química Física e Ingeniería Química, Facultad de Ciencias, University of Alcalá, E-28871 Madrid, Spain; jose.luceno@uah.es; 2 Departamento de Química Orgánica y Química Inorgánica, Facultad de Ciencias, University of Alcalá, E-28871 Madrid, Spain; georgiamaties@gmail.com (G.M.); camino.gonzalez@uah.es (C.G.-A.)

**Keywords:** graphene oxide, functionalization, hexamethylene diisocyanate, dispersion, functionalization degree, morphology, hydrophobicity, thermal stability

## Abstract

Graphene oxide (GO), the oxidized form of graphene, shows unique properties including high mechanical strength, optical transparency, amphiphilicity and surface functionalization capability that make it attractive in fields ranging from medicine to optoelectronic devices and solar cells. However, its insolubility in non-polar and polar aprotic solvents hinders some applications. To solve this issue, novel functionalization strategies are pursued. In this regard, this study deals with the preparation and characterization of hexamethylene diisocyanate (HDI)-functionalized GO. Different reaction conditions were tested to optimize the functionalization degree (FD), and detailed characterizations were conducted via elemental analysis, Fourier-transformed infrared (FT-IR) and Raman spectroscopies to confirm the success of the functionalization reaction. The morphology of HDI-GO was investigated by transmission electron microscopy (TEM), which revealed an increase in the flake thickness with increasing FD. The HDI-GO showed a more hydrophobic nature than pristine GO and could be suspended in polar aprotic solvents such as *N*,*N*-dimethylformamide (DMF), *N*-methylpyrrolidone (NMP) and dimethyl sulfoxide (DMSO) as well as in low polar/non-polar solvents like tetrahydrofuran (THF), chloroform and toluene; further, the dispersibility improved upon increasing FD. Thermogravimetric analysis (TGA) confirmed that the covalent attachment of HDI greatly improves the thermal stability of GO, ascribed to the crosslinking between adjacent sheets, which is interesting for long-term electronics and electrothermal device applications. The HDI-GO samples can further react with organic molecules or polymers via the remaining oxygen groups, hence are ideal candidates as nanofillers for high-performance GO-based polymer nanocomposites.

## 1. Introduction

Graphene (G), an allotrope of carbon like diamond, graphite and fullerenes, has attracted a lot interest in recent years both for fundamental studies and potential applications [1]. It is a flat, atomically thick two-dimensional (2D) sheet composed of sp^2^ carbon atoms arranged in a honeycomb structure. It presents superior electronic, thermal and mechanical properties, very large surface area and the highest electrical conductivity known at room temperature [2]. Its extremely high carrier mobility, broad absorption spectral range, high optical transparency and abundance make very attractive material in various fields ranging from medicine [3] or high-performance composites [4] to chemical sensors [5] and solar cells [6].

Several routes have been reported for the preparation of G, including chemical vapor deposition (CVD) of hydrocarbons onto transition metal surfaces, micromechanical exfoliation of graphite, epitaxial growth on electrically insulating substrates like SiC wafers, electrochemical intercalation, thermal exfoliation or chemical reduction of graphite oxide [7]. However, these approaches lead to a low production yield and are time consuming. Further, due to its hydrophobic nature and strong van der Waals forces between adjacent sheets, it is insoluble in water or common organic solvents and displays poor dispersion in most solvents, which hinders its applications. In this context, graphene oxide (GO), originated from the exfoliation of graphite oxide or the chemical oxidation of G [8], has been studied in much research as an alternative to G. The established advantages of GO in production yield and cost make it an attractive candidate as nanofiller in polymer composites. GO is a water-soluble nanomaterial since it comprises epoxide, hydroxyl and carbonyl groups on the basal planes and carboxylic acids on the edges. Thus, upon sonication in aqueous media, it easily exfoliates and forms stable colloidal suspensions due to its strong hydrophilicity [9]. However, the exfoliation of GO in organic solvents is hindered due to strong hydrogen bonding interactions between adjacent layers. Improving the dispersibility of G-based materials in organic media is crucial from an application viewpoint, hence novel functionalization strategies are pursued. In this regard, the dispersion ability of G and GO in a wide range of solvents has been reviewed [10,11]. If the number of hydrogen bond donor groups in GO is reduced via chemical functionalization, the layers would become less hydrophilic and the strength of interlayer hydrogen bonding will be attenuated, thus allowing for exfoliation in organic solvents. Such functionalization was first demonstrated by Lerf et al. [12], who prepared a series of chemically modified graphite oxide derivatives.

Owed to its π electrons delocalized over the entire 2D network, G is somewhat chemically inert, hence covalent chemical functionalization of pristine G is a challenging task that typically requires reactive species that can form covalent adducts with the sp^2^ carbon structures in G through free radical addition, CH insertion, or cycloaddition reactions [13]. Consequently, most of the research activity has been carried out in the field of chemical modification of GO, one of the hottest topics in nanotechnology that has been explored by a large number of studies [14,15]. In particular, the addition of nucleophilic species, such as amines or hydroxyls, attaches functional groups to GO via formation of amides or esters [14]. The attachment of aromatic or aliphatic amines like polyaniline, pyrrolidine, ethylenediamine, and so forth led to GO derivatives that were homogeneously dispersed in water, *N*,*N*-dimethylformamide (DMF), dimethyl sulfoxide (DMSO), and ethanol, but dispersed poorly in methanol, acetone or propanol and could not be suspended in chloroform, dichloromethane or toluene [16,17]. Polymers such as poly(ethylene glycol), poly(vinyl alcohol) and poly(2-(dimethylamino)ethyl methacrylate) have also been attached to GO via grafting-to or grafting-from approaches [18,19], and the resulting materials showed increased dispersibility in many solvents, including water, methanol, NMP or DMSO.

An interesting approach is the isocyanate functionalization proposed by Stankovich et al. [20]. In their work, reactions between different organic isocyanates and the hydroxyl and carboxyl groups of GO were tested to reduce the amount of hydrogen bonds with donor groups on GO sheets, thus reducing the nanomaterial hydrophilicity. As a result, the isocyanate-treated GO samples could be exfoliated in some polar aprotic solvents such as DMF, after a mild ultrasonication, albeit they could not be dispersed in non-polar solvents. In addition, the epoxy groups of GO can be easily modified through ring-opening reactions, and the resulting materials were well-dispersed in solvents such as water, DMF and DMSO [21].

**Scheme 1:**
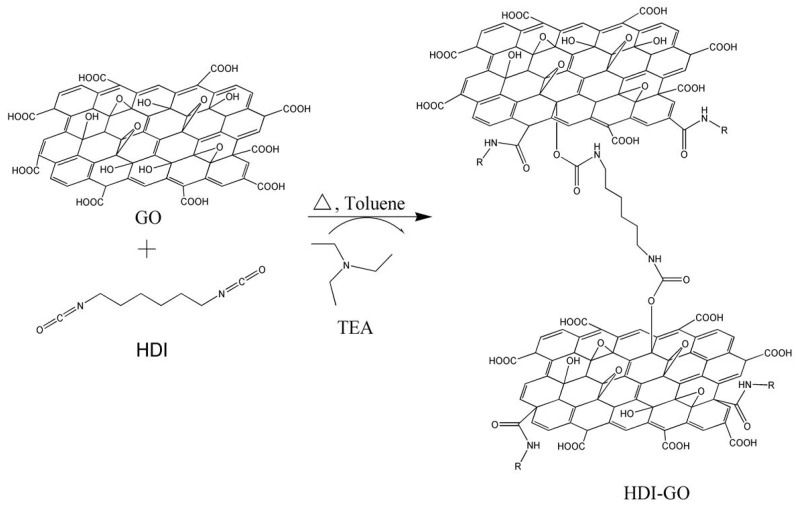
Schematic representation of the synthesis procedure of hexamethylene diisocyanate (HDI)-functionalized graphene oxide (GO).

In most of the aforementioned studies, the resulting GO derivatives showed improved dispersibility both in aprotic and protic polar organic solvents. However, their dispersibility in moderately polar or non-polar solvents was not increased, and this still restricts their use in certain applications like polymeric-based solar cells. In this regard, the aim of the present work is to synthesize and characterize chemically modified GO samples via treatment with hexamethylene diisocyanate (HDI) that can form stable dispersions in a wide range of solvents with different polarities. For such purpose, GO was first prepared using a modified Hummers´ method from flake graphite [8] and then reacted with HDI in the presence of triethylamine (TEA) as a catalyst to yield functionalized HDI-GO nanosheets (Scheme 1).

Different reactions conditions were tested in order to optimize the functionalization degree (FD), and detailed characterizations were conducted to confirm the successful functionalization of HDI on the GO surface. The resulting HDI-functionalized GO derivatives showed a more hydrophobic nature than pristine GO and could be suspended in polar aprotic solvents such as DMF, *N*-methylpyrrolidone (NMP) and DMSO, as well as in some low polar/non-polar solvents like tetrahydrofuran (THF), chloroform and toluene. In addition, the thermal stability was investigated to evaluate the effect of the functionalization on the thermal properties of the nanomaterials. Given that the FD of GO can be controlled by modifying the reaction conditions, it is feasible to synthesize GO sheets with partial HDI functionalization, which could be further subjected to a chemical reaction with other organic molecules or polymers via the residual hydroxyl groups. Further, depending on their FD, their solubility and dispersibility in different solvents can be tailored, which is interesting for the preparation of composites via mixing with polymers that have different solubilities. They could also be used in functional applications including electromagnetic wave absorption materials, electronic devices, field-effect transistors, memory devices, printable electronics, hydrogen storage as well as electrodes in lithium ion batteries, ionic conductors and supercapacitors [13,14]. Besides, due to their more hydrophobic features, they could enable the oil enrichment for the crude oil separation from seawater, and would be useful in coatings, biomedical devices, ultrasensitive sensors and biosensors.

## 2. Materials and Methods

### 2.1. Reagents

Natural graphite was obtained from Bay Carbon, Inc. (Bay City, MI, USA). H_2_SO_4_, KMnO_4_, P_2_O_5_, K_2_S_2_O_8_ and H_2_O_2_ (30 wt% in water) were purchased from Sigma-Aldrich (St. Louis, MO, USA) and used as received. HDI (>99%, C_8_H_12_N_2_O_2_, M_w_ = 168.196 g/mol) was acquired from Acros Organics (Pittsburgh, PA, USA). TEA (>98%, N(CH_2_CH_3_)_3_, M_w_ = 101.193 g/mol) was obtained from Fluka Analytical (Munich, Germany). All the organic solvents were high performance liquid chromatography (HPLC) grade and were purchased from Scharlau S.L. (Barcelona, Spain). Toluene was dried and purified with a MBRAUN solvent purification system (Regensburg, Germany). Ultrapure water was obtained from a Millipore Elix 15824 Advantage 15 UV purification system (Bay City, MI, USA).

### 2.2. Synthesis of GO

GO was prepared using a modified Hummers´ method from flake graphite [3,8]. Briefly, graphite powder, H_2_SO_4_, K_2_S_2_O_8_, and P_2_O_5_, were heated at 80 °C for 5 h. After cooling, deionized water was added to the mixture and it was stirred overnight. The resulting product was then filtered, dried under air and oxidized again via addition of H_2_SO_4_, KMnO_4_ and water in an ice-water bath. Following to dilution with water, excess KMnO_4_ was decomposed by addition of 30 wt% H_2_O_2_ aqueous solution and then 5 wt% HCl aqueous solution. The product was filtered again and purified by repeating the following cycle: Centrifugation, removal of the supernatant liquid, addition of aqueous solution of H_2_SO_4_ (3 wt%)/H_2_O_2_ (0.5 wt%) and bath ultrasonication for 30 min at a power of 140 W. Then, it was washed several times with deionized water and finally vacuum freeze-dried before use.

### 2.3. Synthesis of HDI-Functionalized GO

The whole process was carried out under inert atmosphere of argon in order to avoid contamination during the functionalization reaction. In a typical experiment, GO powder (c.a. 250 mg) was weighed and loaded into a 100-mL round-bottom flask, followed by addition of dried toluene (25 mL) under Ar atmosphere. The suspension was then ultrasonicated in an ultrasonic bath for 2 h; in some experiments, the bath sonication was preceded by probe sonication cycles (5 min on/5 min off, 40% amplitude). The sonication conditions were chosen according to preliminary studies carried out in the group [22]. The GO dispersion was then transferred to a reactor equipped with mechanical agitator, thermometer and reflux condenser. Subsequently, TEA (c.a. 8.75 mL) and HDI (5 mL) were added dropwise via a dropping funnel. The mixture was heated to 60 °C and stirred at 350 rpm overnight under inert atmosphere. The resultant slurry reaction mixture was then poured into methylene chloride to coagulate the product, and finally filtered, washed thoroughly with methylene chloride and dried under vacuum to yield HDI-GO. A schematic representation of the synthesis procedure of functionalized HDI-GO is shown in Scheme 1.

The reaction conditions, namely reaction temperature, reaction time, GO/HDI/TEA ratio, tip/bath sonication cycles and solvent volume, were varied in order to determine their effect on the product yield. The conditions of each experiment and nomenclature of the different HDI-GO samples obtained herein are detailed in Table 1. Similar to pristine GO, the HDI-functionalized GO samples consisted in fine powders. With increasing functionalization degree, the color changed from dark black to metallic grey, indicative of loss of aromatic character.

### 2.4. Instrumentation

A Selecta 3001208 ultrasonic bath and a 24 kHz Hielscher UP400S ultrasonic tip processor (maximum power output of 400 W, Teltow, Germany) equipped with a titanium sonotrode with a diameter of 7 mm and length of 100 mm were used to prepare the GO and HDI-GO dispersions in the different solvents.

Elemental analysis was carried out with a LECO CHNS-932 elemental analyzer (Stockport, UK). Chromatograms were recorded on a gas chromatography/mass spectrometry (GC-MS, Santa Clara, CA, USA) turbo system (5975-7820A) model equipped with a HP-5MS capillary column (30 m × 0.25 mm × 0.25 µm), under the following conditions: Injector temperature 250 °C, detector 150 °C, oven temperature program: 50 °C ramp 5 °C/min until 100 °C, another ramp 10 °C/min until 230 °C (15 min).

Nuclear magnetic resonance (NMR) spectra were recorded using Varian Mercury 300 spectrometer (Santa Clara, CA, USA). Chemical shifts (δ) are reported in ppm and were measured relative by the internal referencing to the CDCl3 (1H).

Fourier-transformed infrared (FT-IR) spectra were recorded with a Perkin Elmer Frontier FTIR spectrophotometer (Waltham, MA, USA) equipped with an Attenuated Total Reflection (ATR) sampling accessory. Spectra were recorded at room temperature, in the wavenumber range of 500–4000 cm^−1^, with an incident laser power of 1 mW and a minimum resolution of 4 cm^−1^. Prior to the measurements, the powder samples were mixed and ground with KBr, and the mixtures were then pressed into a round transparent pellet in a pellet-forming dye.

Room temperature Raman spectra were obtained with a Renishaw Raman microscope (Gloucestershire, UK) equipped with a He-Ne laser (632.8 nm). The laser power at the sample was 1 mW. Three scans were recorded for each sample to reduce the signal-to-noise ratio. For comparative purposes, spectra were normalized to the G band.

Transmission electron microscopy (TEM) images were acquired with a Philips Tecnai 20 FEG (LaB6 filament) electron microscope (Philips Electron Optics, Holland) fitted with an EDAX detector, operating at 200 kV and with 0.3 nm point-to-point resolution. Samples were prepared by re-suspending in an ultrasonic bath about 2 mg of each sample powder in 5 mL of DMF. A drop of each suspension was cast on 200 mesh Lacey copper grids and dried under reduced pressure.

Water contact angle measurements were carried out at room temperature using a Ramé-Hart Model 500 Advanced Goniometer (Succasunna, NJ, USA). Prior to the measurements, the samples were dispersed in DMF and spin coated onto silane-functionalized glass substrates. A drop of Milli-Q water was deposited placed on the sample surface and the evolution of the droplet shape was recorded with a CCD video camera. DROPimage Advanced v2.4 analysis software was used to determine the contact angle. For each sample, the reported value is the average of the results obtained on three droplets.

The functionalization degree of HDI-GO and thermal stability of the samples was determined by thermogravimetric analysis (TGA) tests under a nitrogen atmosphere using a TA-Q500 thermobalance (Barcelona, Spain) coupled to a mass spectrometer, at a heating rate of 10 °C/min, from 100 to 600 °C.

## 3. Results and Discussion

### 3.1. Reaction Mechanism and Functionalization Degree

**Scheme 2:**
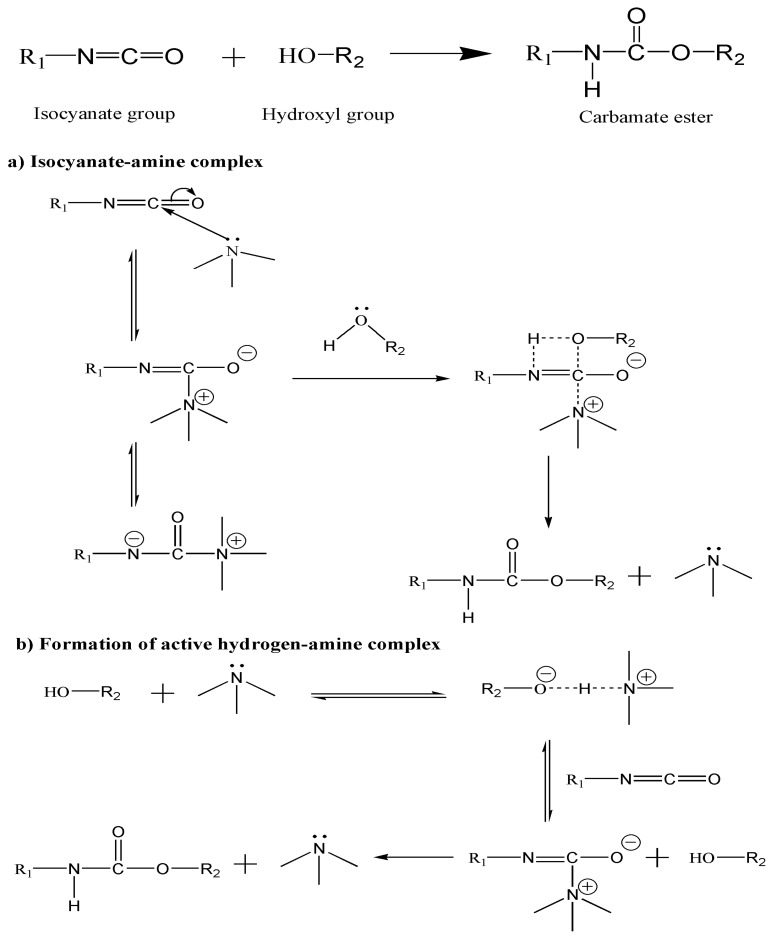
Mechanism of the reaction between an isocyanate and a hydroxyl group to form a carbamate ester catalyzed by ternary amines: (**a**) Formation of an isocyanate-amine complex; (**b**) formation of active hydrogen-amine complex.

Organic isocyanates can react with both the edge carboxylic acid and surface hydroxyl or epoxide functional groups of GO through formation of amides or carbamate esters, respectively [23]. Isocyanate reactions are complicated owed to several reasons: (1) Competing side reactions, (2) reversible reaction steps, and (3) catalytic effect of reactants and products. Thus, it turns out to be quite difficult to establish a reaction mechanism and a kinetic model. The reactivity of isocyanate vs. different functional groups follows the order: Primary amine > secondary amine > hydroxyl > acid > anhydride > epoxide [24]. The reaction between an alcohol and an isocyanate group to form a carbamate ester is moderate, hence is usually catalyzed by bases, mainly tertiary amines like TEA [25]. The catalytic activity of amines is attributed to the presence of a lone pair on the nitrogen atom. Several mechanisms regarding the catalytic activity of amines can be found in the literature [25,26,27], the most important ones being the formation of an isocyanate-amine complex or an active hydrogen-amine complex (Scheme 2).

The first mechanism (Scheme 2a), proposed by Baker and Holdsworth [27] involves the reversible nucleophilic attack on the carbon atom of the isocyanate by the amine to form a complex in which the nitrogen of the NCO group is activated, making easier the reaction with the lone electrons of the oxygen of the alcohol. Then, the complex is decomposed, forming the carbamate ester and regenerating the base. Another mechanism proposed by Farkas and Strohm [25] involves the activation of the alcohol via hydrogen bonding with the amine, resulting in the formation of a complex that subsequently reacts with the isocyanate group (Scheme 2b).

**Figure 1:**
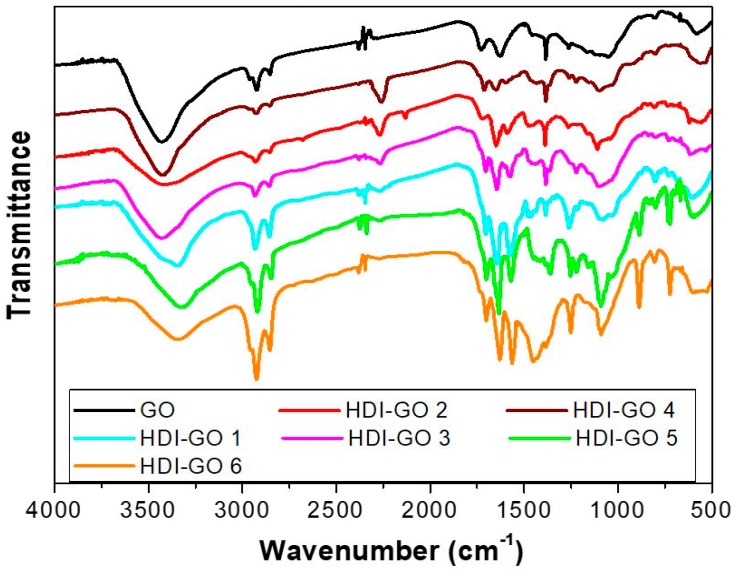
Fourier-transformed infrared (FT-IR) spectra of raw GO and the different HDI-GO samples.

Isocyanates can also react with carboxylic acids via an amidation reaction, albeit the yield in conventional organic solvents is typically low [28]. Besides, the side reaction of isocyanates with water results in amine formation and CO_2_ gas release. The reaction is exothermic and is also catalyzed by tertiary amines. It has been demonstrated that the isocyanate reactivity with water and primary hydroxyl group is comparable, and more than double that with carboxylic acids [24]. Isocyanates also react with amines to give ureas. On the other hand, the epoxide functionality is the least reactive; the reaction between isocyanate and epoxide to form carbamate ester linkages is unlikely and only takes place in the presence of special catalysts [29]. From all the above discussion, and taking into account the IR spectra of the different HDI-GO samples (Figure 1), which show the appearance of an intense absorption peak at ~1710 cm^−1^ ascribed to the C=O stretching vibration of carbamate esters [20], as will be discussed in detail in the following section, it can be concluded that the formation of carbamate esters via reaction of HDI with surface OH groups of GO is the most likely.

Given that HDI possesses two reactive isocyanate groups, it can act as a crosslinking agent, joining adjacent GO layers (Scheme 1). A very high level of crosslinking is not desirable, since it can make the exfoliation of the functionalized GO into individual sheets difficult, thus hindering subsequent reactions with other organic molecules or polymers via the remaining oxygen groups. The crosslinking between the GO sheets that are randomly covalently bonded by HDI to each other combined with the higher hydrophobic character of the HDI-GO compared to GO will influence their physical properties (i.e., solubility, polarity, thermal stability, etc.), as will be discussed in the following sections.

The results obtained from elemental analysis of neat GO and the different functionalized samples are collected in Table 2. Assuming that the formation of carbamate esters through reaction of HDI with the OH surface functional groups of GO is the unique reaction path, the nitrogen-to-carbon atomic ratio can be used to roughly estimate the functionalization degree (FD). In particular, FD can be expressed as moles of carbamate ester unit incorporated per mol of carbon atoms of GO, and the calculated values are tabulated in Table 2. It should be noted that these values correspond to a lower bound of functionalization; in the case of amide formation, which seems less feasible according to the IR spectra (Figure 1) since there is no peak at around 1680–1650 cm^−1^ related to the amide I band, the FG would be higher due to a loss of carbon (as carbon dioxide) from the isocyanate reagent.

To understand the data obtained, the factors that influence the chemical kinetics of a reaction, namely reactant concentrations, temperature, physical states and surface areas of reactants, solvent and catalyst concentration and properties, and so forth, have to be taken into account. The kinetics and mechanism of tertiary-amine catalyzed reaction between isocyanates and primary alcohols have been investigated by several authors [27,30,31,32,33], and it is commonly accepted that the reaction proceeds by competitive consecutive second order reactions through a carbamate ester-isocyanate intermediate; besides, the reaction rate was found to be directly proportional to the concentration of each reactant and the amine catalyst [32].

Given that it is not possible to determine the molarity of GO because it is a non-stoichiometric compound and it has no specific molecular weight, it was not feasible to reproduce the concentration ratios of reactants and catalyst previously reported for the reactions of isocyanates with alcohols. Therefore, the first trial (HDI-GO 1) was carried out with GO:HDI:TEA weight ratios of 1:1:1, leading to a FD of around 12% (Table 2, entry 2). Surprisingly, the increase in the amount of HDI reactant and TEA catalyst (HDI-GO 2, with GO:HDI:TEA weight ratios of 0.5:1:1) results in a drop in FD (Table 2, entry 3). The reaction extent is likely limited by the number of hydroxyl groups on the GO surface available for reaction. Hence, the increase in the HDI concentration and TEA does not lead to a higher FD. It is well known that the molecules of TEA do not associate with each other albeit form hydrogen bonds with alcohols to some extent. Since TEA acts as a catalyst, when its concentration is increased, the excess of molecules likely interact with the OH surface groups of GO via hydrogen bonding, hence competing with or hindering the reaction between the hydroxyl moieties and HDI, consequently the reaction yield does not increase or even decreases. This is consistent with the observations reported earlier by other authors [31,33], who found that the reactivity of the OH groups depended on whether they were free or hydrogen bonded, because the activation energies of hydrogen bonded alcohols for carbamate ester formation were considerably higher than those of free alcohols. Further, Sato [31] found a decrease in the reaction rate with increasing the amount of reactants. It was suggested that when the concentration of isocyanate was doubled, it spontaneously reacted with the moisture contained in the solvent or present in the environment and with the carbamate to produce a small amount of the corresponding urea, thus the reaction yield was reduced. In fact, alkyl-alkyl urea has been reported to be a major byproduct in the reactions of aliphatic isocyanates with alcohols [34]. However, in this study the synthesis of HDI-GO was carried out under an Ar atmosphere and with dried toluene, hence the formation of urea byproduct, in particular *N*,*N*’-dihexylurea, is not likely. Further, under some circumstances in the presence of amine catalysts, isocyanates can form dimers, trimers or polymerize [35] by linear polymerization and condensation with elimination of carbon dioxide to form uretidinone, isocyanurate, and carbodiimide. In particular, traces of *N*,*N*´-dihexylcarbodiimide could be formed by decomposition of HDI dimmers [35].

To corroborate the absence of urea and carbodiimide byproducts, the toluene removed during the filtration stage was analyzed by gas chromatography/mass spectrometry and RMN techniques. The chromatogram showed only one compound and the mass spectra revealed that this peak corresponded to the initial product. The ^1^H NMR spectrum exhibited just three peaks related to protons of OCNCH_2_ [3.31 ppm, t], OCNCH_2_CH_2_ [1.70–1.55 ppm, m] and OCNCH_2_CH_2_CH_2_ [1.49–1.31 ppm, m]; these chemical shifts are in perfect agreement with those of the initial HDI, which confirms the lack of byproducts in the synthesis of HDI-GO.

Regarding the reaction carried out for 48 h (HDI-GO 3), a slight fall in FD is found (Table 2, entry 4) compared to the initial trial. Lu et al. [24] reported that the conversion yield of the reaction between an isocyanate and an alcohol crosses over at longer reaction times, suggesting partial reversibility and equilibrium. Similarly, Sato [31] found that at high concentrations of both reactants, the reaction rate decreased rapidly with increasing reaction time. These facts could explain the small drop in FD at very long reaction times.

On the other hand, the rise in the reaction temperature (HDI-GO 4) also resulted in a lower FD (Table 2, entry 5), which was only about 3%. This is consistent with the results reported previously for the reaction of n-alcohols with phenyl isocyanate, where the rate of the reaction vs. 1/T followed a straight line [30]. This behaviour could be rationalized considering that the reaction between an alcohol and an isocyanate is exothermic, hence when the temperature is increased, the yield of products is decreased. Besides, it has been reported that at elevated temperatures the reaction becomes rapidly reversible [36], giving isocyanate and alcohol. This is in agreement with the IR spectrum of HDI-GO 4, which shows a very intense O–H stretching peak at 3430 cm^−1^ and N=C=O stretching of the isocyanate groups at 2260 cm^−1^. These peaks gradually drop in intensity with increasing FD, indicative of a lower extent of the reverse reaction.

Another factor that could influence the reaction yield is the solvent volume. Given that the functionalization reaction should take place on the surface groups of GO, the HDI and TEA have to diffuse from the bulk solution to the GO surface, and the diffusion is typically the slowest stage that controls the overall reaction rate. The solvent facilitates the diffusion of the reactant and catalyst towards the nanomaterial surface; if the solvent volume is too low, these cannot effectively diffuse, and the reaction becomes saturated. Therefore, low solvent volumes likely limit the reaction yield. Further, the solvent molecules arrange around the reactants forming solvent cages. This “cage effect” hinders the separation of the reactant molecules and easily absorbs the excess energy of products, thus favouring product formation [37]. The increased number of collisions caused by a stronger solvent cage effect would increase the reaction rate.

To test this hypothesis, further experiments were carried out with double volume of solvent (HDI-GO 5 and HDI-GO 6). Besides, to enlarge the surface area of GO available for the reaction, it was subjected to sonication with an ultrasonic probe, a process that effectively induces the exfoliation of the nanomaterial into thinner sheets. However, this procedure generates defects in the GO flakes: Point defects on the basal plane and edge defects, which may have detrimental effects on the nanomaterial properties, and the number of defects rises with increasing ultrasonication time [38]. Therefore, an optimum balance between level of exfoliation and defect density has to be attained. For such purpose, the probe sonication was combined with bath ultrasonication, which is less aggressive. The probe sonication conditions (5 min at 40% amplitude) were chosen according to previous studies carried out in the group [22]. By only applying one probe sonication cycle (HDI-GO 5), a FD of about 17% was attained (Table 2, entry 6). Another experiment was performed by applying three probe sonication cycles with a 5 min break between cycles, leading to a FD close to 18% (Table 2, entry 7). It is therefore confirmed that the longer the sonication with the probe, the better the level of GO exfoliation, the larger the surface area on which collisions can occur, hence the most effective the reaction is. Nonetheless, the differences in the extent of the reaction between HDI-GO 5 and HDI-GO 6 are relatively small, hinting that a saturation level has been attained and the FD has been leveled off, hence these were taken as the optimum ones.

### 3.2. FT-IR Study

The chemical changes that occurred upon treatment of GO with HDI were monitored by FT-IR spectroscopy, since both GO and the derivatives display characteristic IR spectra (Figure 1). As mentioned earlier, GO contains epoxide and hydroxyl functional groups on both sides of its basal plane and carboxyl moieties at the edge sites. The most characteristic features in the IR spectrum of GO are the strong broad band centered at ~3430 cm^−1^ corresponding to the O–H stretching vibrations, the peaks at around 2925 and 2845 cm^−1^ attributed to sp^2^ and sp^3^ C–H stretching bands produced at defects sites of the graphene network, the peak at ~1730 cm^−1^ arising from the C=O stretching of the carboxylic acid groups, the band at 1620 cm^−1^ assigned to the aromatic C–C stretching, that at ~1400 cm^−1^ corresponding to the O–H deformation [39] and the C–OH stretching at 1260 cm^−1^ [3].

Upon treatment with HDI, the intensity of the O–H stretching band was reduced, decreasing gradually with increasing FD, and also shifted towards lower wavenumbers, due to the overlapping with the N–H stretching vibrations of the carbamate groups. Further, the peaks at 2845 and 2925 cm^−1^ originating from symmetrical and asymmetrical stretching vibrations of –CH_2_– became more intense upon raising FD, due to an increased number of methylene chains arising from the HDI. A new band appeared in the samples with low FD at 2280 cm^−1^ ascribed to unreacted N=C=O group [40], suggesting the absorption/intercalation of the organic isocyanate between the GO flakes. However, this band can hardly be detected in the HDI-GO 5 and HDI-GO 6 samples, corroborating that the –N=C=O groups of HDI reacted completely with the hydroxyl groups of GO. Further, the C=O stretching appearing at 1730 cm^−1^ in pristine GO is hidden by a new peak at ~1710 cm^−1^ ascribed to the C=O stretching of the carbamate ester groups [20]. This peak becomes more intense and shifts gradually towards lower wavenumbers with increasing FD.

Besides, new intense bands can be observed at ~1648 and 1580 cm^−1^; the first can be assigned to the coupling of the C=O stretching with in-phase N–H bending and the second to the coupling of the N–H bending with the C–N stretching vibration [40]. These bands could originate from either amide or carbamate ester groups, although in the first case they typically appear at 1660 and 1550 cm^−1^, whilst for carbamate esters the bands are closer together due to the stronger π-π interaction between the carbonyl group and the nitrogen lone pair electrons, and the amide II band appears at higher frequency [41]. These two peaks also turn out to be stronger and move to lower wavenumbers with increasing FD, which could be indicative of increased H-bonding interactions between carbamate esters closely located [42]. Other bands become visible at around 1110 cm^−1^, likely related to –C(=O)–O and C–N stretching vibrations of the carbamate groups, at 885 cm^−1^ attributed to C–H out-of-plane bending vibrations of substituted aromatic rings and at around 720 cm^−1^, ascribed to the rocking of the methylene groups of HDI. On the basis of all the aforementioned observations, it can be concluded that GO was successfully functionalized with the organic HDI reactant and that the functionalization route via carbamate ester formation predominates.

### 3.3. Water Contact Angle Measurements

**Figure 2:**
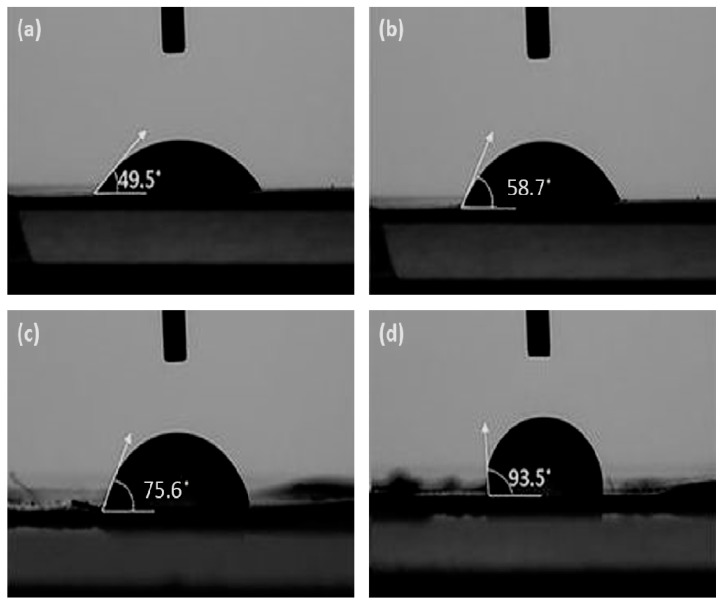
Water contact angle measurements of GO (**a**); HDI-GO 2 (**b**); HDI-GO 1 (**c**); and HDI-GO 6 (**d**).

Water contact angle (CA) measurements were performed to evaluate the hydrophobic or hydrophilic character of the synthesized HDI-GO. In general, surfaces with contact angles ≥90° are regarded as hydrophobic, or in other words water-repellent. Figure 2 compares the contact angles for GO (a), HDI-GO 2 (b), HDI-GO 1 (c) and HDI-GO 6 (d), and the CA values for all the HDI-GO samples synthesized herein are summarized in Table 2. Raw GO is highly hydrophilic (CA ~50°) due to its large number of surface oxygen-containing groups that lead to an immediate absorption of water and swelling of the sample. A gradual increase in CA is found upon increasing FD, indicating lower level of hydrophilicity (wettability), ascribed to the decrease in the number of surface hydroxyl groups due to their reaction with HDI and the higher degree of crosslinking between the GO flakes that reduces the swelling capacity [43]. The HDI-GO samples with FD ≤12% show CA values in the range of 54–75°, hence can be still regarded as hydrophilic like the parent GO. It is interesting to note that the most favorable CA values for protein adsorption and cell adhesion have been reported to be in the range of 40–70° [44], which suggests that the derivatives with low functionalization degree could be suitable for biomedical applications. On the other hand, the HDI-GO 5 and HDI-GO 6 samples have a CA ≥90°, hence are hydrophobic in nature. Overall, it is found that the functionalization reaction with HDI leads to an increased surface hydrophobicity and reduces the hydration of the remaining hydroxyl groups.

### 3.4. Solubility Tests

**Figure 3:**
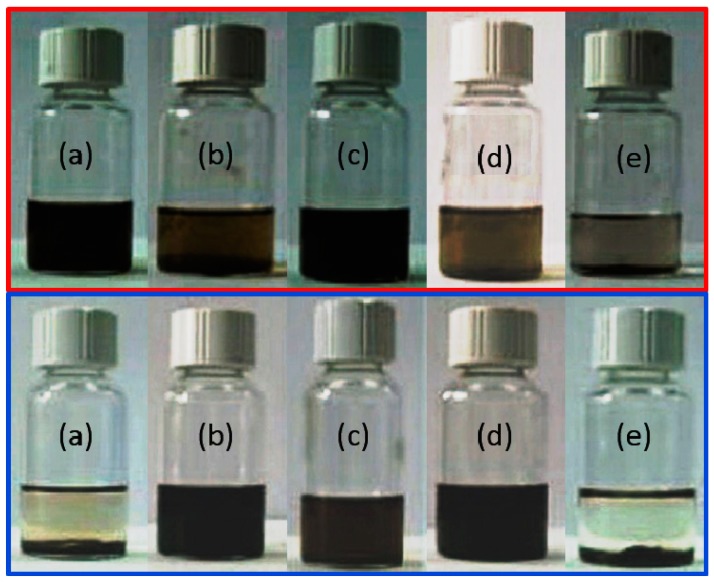
Photographs of the dispersions of GO (**top**) and HDI-GO 6 (**bottom**) in different solvents: (**a**) Water; (**b**) DMF; (**c**) NMP; (**d**) toluene; (**e**) n-hexane.

As detailed in the introduction, the stability of HDI-GO in a range of solvents is a critical point for the preparation of dispersions to be applied in various fields. In this context, the solubility/dispersibility of the functionalized samples was investigated in water and organic solvents of different nature, and directly compared with that of pristine GO. The following organic solvents were tested: Acetone, methanol, ethanol, 2-propanol, THF, DMF, NMP, DMSO, chloroform, toluene, n-hexane and n-pentane. For solubility tests, a small quantity of GO or HDI-GO (~0.5 mg) was added to a given volume of solvent (~1 mL) in a test tube and gently stirred with a glass stirring rod for a few min. To check the dispersibility, the samples were sonicated in an ultrasound bath for 10 min. The results obtained from the solubility tests are summarized in Table 3, and representative photographs of the GO and HDI-GO 6 dispersions in water, DMF, NMP, toluene and n-hexane are shown in Figure 3.

Pristine GO is completely soluble in water, propanol, THF and NMP (Table 3, entry 1), in agreement with the results reported previously [45]. However, despite its high oxygen content (~52%, Table 1), it is only partially soluble in polar protic solvents, such as methanol or ethanol (Table 3, entry 8). This indicates that the solvent polarity is not the only issue controlling the solubility. Other factors such as the solvent dipole moment, its surface tension and the Hansen solubility parameters play a key role on the solubility behaviour [46]. Thus, the best solvents exhibit high dipole moment values (i.e., 1.85, 1.66 and 3.75 for water, propanol and NMP [46]) and a surface tension close to the reported surface energy of GO (~62 mN/m [47]). It is worth mentioning that GO showed low solubility but stable dispersibility in non-polar solvents, like toluene or chloroform, while was insoluble in hexane or pentane.

In contrast to neat GO, the HDI-GO samples were hardly soluble in water and polar protic solvents, and the solubility decreased with increasing FD. However, upon a short ultrasonication treatment, they swelled and formed colloidal dispersions in polar aprotic solvents such as DMF, NMP and DMSO, and the dispersibility improved with increasing FD. The HDI-GO samples with higher FD showed better dispersibility in DMF and DMSO than the parent GO, and were stable for a few weeks. Owed to their higher hydrophobic character, they could also be more easily suspended in low polar solvents like THF or non-polar ones such as chloroform and toluene (Figure 3d). The reduction of hydrogen bonds triggered by the functionalization with HDI renders the GO surfaces more hydrophobic, and the strength of hydrogen bonding between the HDI-GO layers is weakened, thus allowing for improved dispersion in organic solvents. Overall, DMF and DMSO were chosen as optimum solvents to prepare HDI-GO dispersions for further reaction with conductive polymers.

### 3.5. TEM Analysis

**Figure 4:**
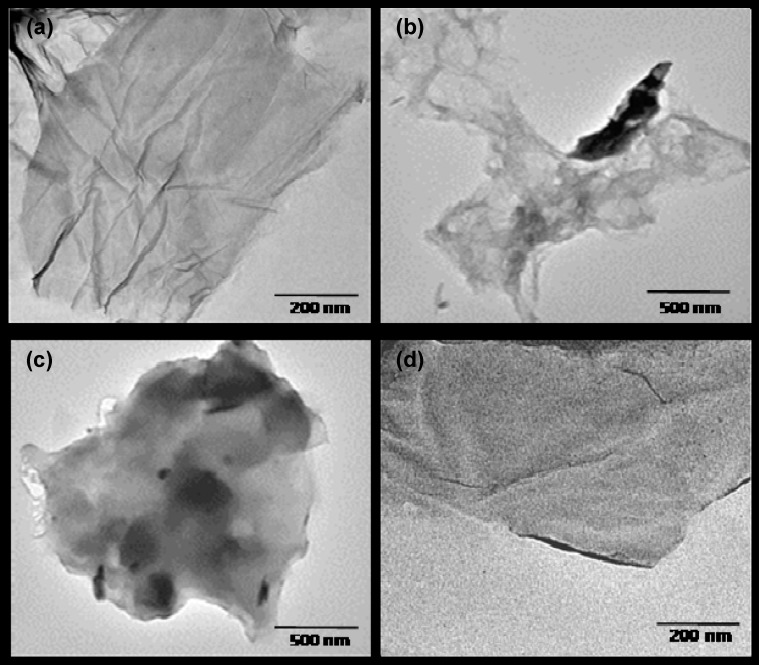
Typical transmission electron microscopy (TEM) images at different magnifications of GO (**a**); HDI-GO 2 (**b**); HDI-GO 5 (**c**); and HDI-GO 6 (**d**).

The surface morphology of neat GO and the different functionalized samples was examined by TEM, and typical images at different magnifications of GO, HDI-GO 2, HDI-GO 5 and HDI-GO 6 are compared in Figure 4.

The thickness of a single monolayer graphene nanosheet has been reported to be about 0.34 nm [2], and a GO sheet is expected to be much thicker due to the presence of oxygen-containing functional groups attached on both sides of the flakes. Pristine GO displays a transparent and pleated sheet structure (Figure 4a), with wrinkled and highly flexible flakes of a thickness ≤10 nm. The sample appears essentially homogeneous and uniform, comprising a few oxidized graphene layers.

Conversely, the HDI-GO samples seem more heterogeneous, and present a relatively fuzzy morphology due to the grafting of the HDI chains onto the nanomaterial surface. Apparently, the darker areas in the images correspond to the alkyl chains of the organic diisocyanate, since the number of dark areas increases with increasing FD. This is consistent with the results reported previously by other authors who grafted alkyl segments onto G via reaction with surfactant molecules [48,49] or polymeric chains as described elsewhere [50]. According to those studies, the grafted alkyl chains are extended in solution due to their solubility in the solvent; nonetheless, upon drying the alkyl segments collapse onto the surface of the GO sheets, forming nanosized domains that likely correspond to the observed dark spots.

In the samples with higher FD, a morphology of fluffy and smooth surfaces in a stack state can be observed (Figure 4c), ascribed to the alkyl chains irregularly arranged on the GO surface, particularly at the wrinkles and edges that are more reactive owed to the lattice defects. The surface foldings cannot be observed in these functionalized samples, likely due to the wrapping effect of the organic moieties attached to the GO which, thus, enshroud these wrinkles. Besides, the surface of the HDI-GO samples is coarser than that of GO, with some visible defects, albeit retaining the structural integrity of the graphene framework, and the flake thickness increases with increasing FD.

It should be noted that GO is non-homogeneously covered by the HDI chains in the derivatives with low FD (Figure 4b), whereas the HDI-GO 6 shows a much more uniform coating (Figure 4d), in agreement with its higher extent of functionalization. The GO surface appears fully covered by a spotted dark pattern, suggesting that a complete coverage has been attained; hence it would be difficult to obtain a higher FD.

### 3.6. Raman Spectra

**Figure 5:**
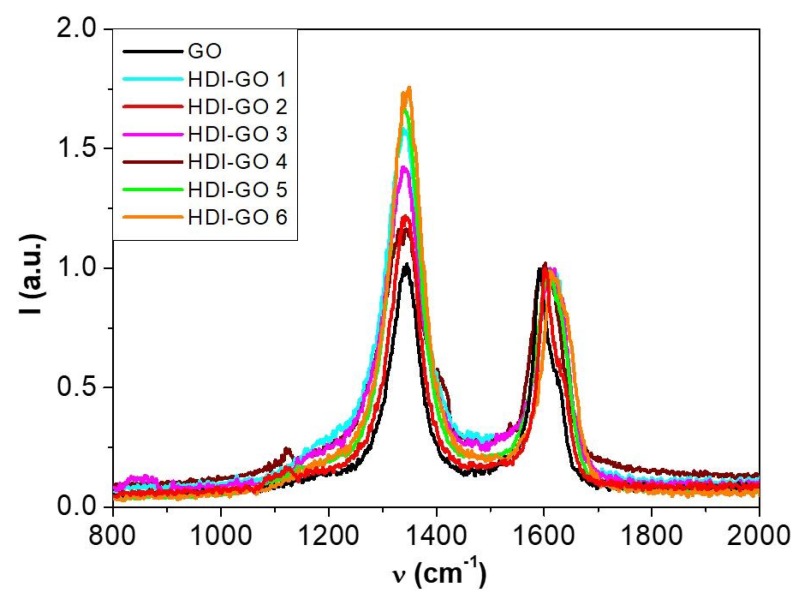
Raman spectra of GO and the different HDI-GO samples.

Raman spectroscopy was carried out to verify the structural changes in GO upon reaction with HDI, and the spectra of the different samples are compared in Figure 5. The most important features in the Raman spectrum of graphene materials are the disorder induced D band at 1345 cm^−1^ that indicates the level of structural disorder and the tangential G band at ~1595 cm^−1^ arising from in-plane displacements in the graphene sheets.

The D to G band intensity ratio (I_D_/I_G_) is widely used to obtain quantitative information about the amount of defects in graphene materials: The higher the ratio, the higher the disorder [51]. The calculated I_D_/I_G_ data for raw GO and the HDI-GO samples are summarized in Table 4, along with the position of the G band. A steady increase in this ratio is observed with increasing FD, from about 1 for GO (Table 4, entry 1) to 1.75 for the derivative with the highest FD (Table 4, entry 7), which demonstrates a decrease in the structural order, that is, in the size of the in-plane sp^2^ domains, owed to the covalent grafting of the HDI chains onto the GO surface. An analogous trend of I_D_/I_G_ increase has been reported upon covalent functionalization of GO with other organic molecules such as 4-aminobenzenesulfonic acid [52] or polymers like poly(N-isopropylacrylamide) (PNIPAM) [53], hinting that the functionalization process results in graphitic domains that are smaller than those of GO.

It can also be observed from the comparison of the Raman spectra that upon increasing FD the G-band of GO is broadened and upshifted to higher wavenumbers, by up to 22 cm^−1^ for HDI-GO 6 (Table 4, entry 7) compared to pristine GO (Table 4, entry 1). The change in the position of the G band is typically associated with a modification of the electronic structure of the carbon nanomaterial [54], being shifted to higher frequencies in the presence of electron-acceptor groups. Albeit the electron distribution of the isocyanate group makes it either an electron donor or electron acceptor, in the reaction performed herein it seems to act as an electron-acceptor (Scheme 2), thus causing a blue shift of the G band of GO. The blue shift could also be indicative of the damage of the hexagonal network of carbon atoms, thus a marker of the defect density, since the position of this G band has been reported to shift almost linearly with increasing the concentration of defects in the graphene layer [55]. Besides, upshifts in the G band of graphene-based materials have been reported in the presence of polymers [22] and surfactants [51], ascribed to intense polymer (or surfactant)-graphene interactions. In addition, the G mode peak position depends on the number of GO layers: It shifts to higher frequency as the number of GO layers decreases [55]. Therefore, the steady upshift found here upon increasing FD suggests a reduction in the degree of exfoliation of the GO flakes due to the constraints imposed by the crosslinks between the nanosheets, and consequently, could be indicative of a transition from few-layered to multi-layered GO. This is in agreement with TEM analysis (Figure 4), which revealed a raise in layer thickness with increasing FD.

### 3.7. Thermal Stability

**Figure 6:**
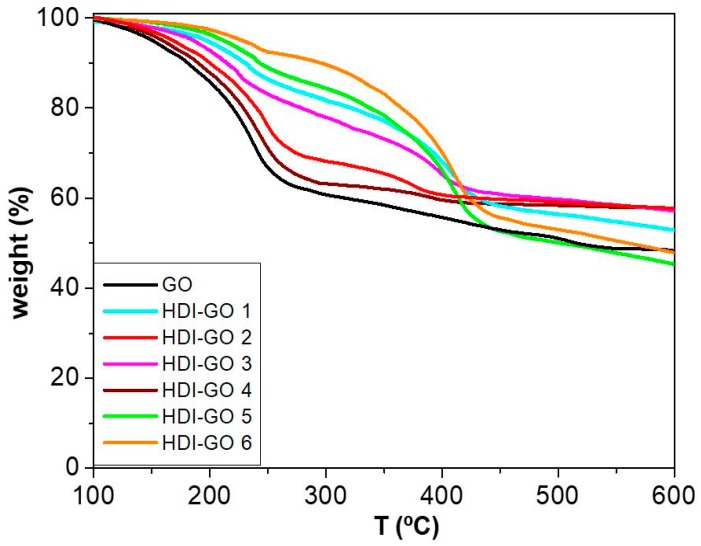
TGA curves under a nitrogen atmosphere of GO and the synthesized HDI-GO.

Figure 6 compares the TGA thermograms under a nitrogen atmosphere of neat GO and the different HDI-GO samples. The pristine nanomaterial shows a one-step degradation process that starts (T_i_) at 124 °C and exhibits the maximum rate of weight loss (T_max_) at ~235 °C (Table 4, entry 1), with about 37% weight loss below 250 °C. This major weight loss is ascribed to the decomposition of its surface oxygen-functional groups, namely epoxide, hydroxyl and carboxylic acid, as revealed by the IR spectrum. Besides, a small gradual mass loss is found above 250 °C, attributed to the removal of additional functional groups [3]. In contrast, the HDI-GO samples display two major stages. The first stage is analogous to that found for neat GO, and should be due to the pyrolysis of the residual oxygen functional groups. The weight loss of this step, that is, the amount of surface oxygen moieties in the functionalized GO, gradually decreases with increasing FD, which can be explained considering that the higher the FD of the HDI-GO samples, the greater the number of oxygenated functional groups of GO that have reacted with the isocyanate groups of HDI, hence the lower the number of residual oxygenated groups on the GO surface that decompose during this stage. Therefore, this decreasing trend in weight loss with increasing FD corroborates the larger extent of the functionalization reaction, in agreement with the results from FT-IR analysis (Figure 1).

The second stage corresponds to the thermal decomposition of covalently grafted HDI on the surface of GO, as previously reported for other alkyl-modified GO derivatives [50], and its mass loss rises with increasing the degree of grafting. From this stage the extent of covalent functionalization or FD of each HDI-GO sample was determined, and the results are collected in Table 4. The values obtained follow exactly the same trend to those derived from elemental analysis, albeit the data from TGA thermograms are systematically higher (i.e., about 1.5 fold larger), which can be explained considering the different meaning of the FD provided by each technique.

Besides, the characteristic degradation temperatures, namely T_i_, T_max_ and the temperature of 10% weight loss (T_10_), grow steadily as the extent of functionalization increases (Table 4), which could be due to the higher degree of crosslinking between the nanosheets, as previously reported for chitosan/GO crosslinked composites [56]. Increasing the amount of crosslinking causes less of the sample to decompose in the first step. Thus, T_i_ increased by about 80 °C from neat GO (Table 4, entry 1) to the HDI-GO 6 sample (Table 4, entry 7); since HDI has two isocyanate groups, it can act as a crosslinking agent between the GO sheets, which is reflected in higher decomposition temperature. The TGA results confirm that the covalent attachment of HDI greatly improves the thermal stability of GO, which could be interesting for a variety of applications including long-term electronics or electrothermal devices.

## 4. Conclusions

HDI-GO derivatives with different functionalization degrees have been synthesized following a two-step approach: Firstly, GO was prepared using a modified Hummers´ method from graphite, and secondly GO was treated with HDI in the presence of a TEA catalyst to yield the modified nanomaterial. The FT-IR and Raman spectra corroborated the success of the reaction and that the functionalization route via carbamate ester formation predominated. The increase in the amount of HDI reactant and TEA catalyst, as well as the increase in the reaction time or temperature resulted in a drop in the extent of functionalization, whilst the combination of probe and bath sonication with higher solvent volumes led to the highest functionalization degrees. The HDI-GO displayed a more hydrophobic character than raw GO and could be dispersed in polar aprotic solvents such as DMF, NMP and DMSO as well as in some low polar/non-polar solvents like THF, chloroform and toluene. Further, the dispersibility in these solvents improved as the functionalization degree increased. The surface morphology of the samples was analyzed by TEM, and the images showed a raise in layer thickness with increasing extent of functionalization. TGA study revealed that the covalent grafting of HDI enhances the thermal stability of GO, ascribed to the crosslinking between neighbouring sheets. This thermal stability improvement is highly attractive for applications such as long-term electronics and electrothermal devices. Future work will focus on the reaction of HDI-GO with other organic molecules or polymers via the remaining oxygen groups, in order to develop high-performance GO-based nanocomposites.

## Figures and Tables

**Table 1:** Nomenclature and reaction conditions for the synthesis of the different HDI-GO samples.

Entry	Sample	Reaction Time (h)	Reaction Temperature (°C)	GO/HDI/TEA Weight Ratio	Tip/Bath Sonication Time (min)	Solvent Volume (mL)
1	GO	-	-	-	-	-
2	HDI-GO 1	12	60	1/1/1	0/120	25
3	HDI-GO 2	12	60	0.5/1/1	0/120	25
4	HDI-GO 3	48	60	1/1/1	0/120	25
5	HDI-GO 4	12	90	1/1/1	0/120	25
6	HDI-GO 5	12	60	1/1/1	5/120	50
7	HDI-GO 6	12	60	1/1/1	5 + 5 + 5 */120	50

* 3 probe sonication cycles with 5 min of break between cycles

**Table 2:** Elemental analysis data, functionalization degree (FD) and water contact angle (CA) for neat GO and the different HDI-GO samples.

Entry	Sample	C (%)	O (%)	H (%)	N (%)	S (%)	FD * (%)	CA (°)
1	GO	41.93	51.96	3.44	0	2.67	0	49.5
2	HDI-GO 1	53.08	35.70	4.22	6.02	0.98	12.28	75.6
3	HDI-GO 2	47.38	44.75	3.83	2.49	1.55	5.08	58.7
4	HDI-GO 3	50.36	40.16	4.01	4.46	1.01	9.10	68.6
5	HDI-GO 4	45.98	46.97	3.67	1.53	1.85	3.12	54.3
6	HDI-GO 5	55.49	31.07	4.50	8.43	0.51	17.20	89.8
7	HDI-GO 6	56.04	30.09	4.55	8.88	0.44	18.13	93.5

* moles of carbamate ester unit incorporated per mol of carbon atoms of GO

**Table 3:** Solubility of GO and the different HDI-GO samples in different solvents.

**Entry**	**Sample**	**Water**	**DMF**	**NMP**	**DMSO**	**CH_3_Cl**	**Toluene**	**THF**
1	GO	S	PS/SS	S	PS/SS	PS/SS	PS/SS	S/PS
2	HDI-GO 1	I	PS	PS	PS	I	I	SS
3	HDI-GO 2	PS	PS/SS	S	PS	SS	SS	PS
4	HDI-GO 3	SS	PS	PS	PS	I	I	SS
5	HDI-GO 4	PS	PS/SS	S	PS/SS	SS	SS	PS
6	HDI-GO 5	I	PS	PS	PS	I	I	I
7	HDI-GO 6	I	PS	PS	PS	I	I	I
**Entry**	**Sample**	**Acetone**	**Pentane**	**Hexane**	**Methanol**	**Ethanol**	**Propanol**	**FD**
8	GO	I	I	I	SS	PS	S/PS	0
9	HDI-GO 1	PS	I	I	I	I	SS	12.28
10	HDI-GO 2	I	I	I	PS	PS	PS	5.08
11	HDI-GO 3	SS	I	I	SS	SS	PS	9.10
12	HDI-GO 4	I	I	I	PS	PS	S/PS	3.12
13	HDI-GO 5	PS	I	I	I	I	I	17.20
14	HDI-GO 6	PS	I	I	I	I	I	18.13

S: Soluble; PS: Partially soluble; SS: Slightly soluble; I: Insoluble

**Table 4:** I_D_/I_G_ ratio and position of the G band obtained from the Raman spectra as well as thermogravimetric analysis (TGA) data of the different samples.

Entry	Sample	T_i_ (°C)	T_10_ (°C)	T_max_ I/II (°C)	FD (%)	I_D_/I_G_	G (cm^−1^)
1	GO	124.1	180.6	235.0	-	1.01	1595
2	HDI-GO 1	165.4	230.7	250.5/396.2	21.9	1.57	1611
3	HDI-GO 2	141.3	199.5	248.6/374.6	7.6	1.22	1603
4	HDI-GO 3	148.6	213.6	245.6/390.6	16.1	1.40	1609
5	HDI-GO 4	130.2	189.2	241.9/385.5	4.5	1.15	1600
6	HDI-GO 5	174.5	242.8	250.9/404.2	29.2	1.66	1613
7	HDI-GO 6	186.3	288.3	249.7/406.6	30.8	1.75	1617

T_i_: Initial degradation temperature at 2% weight loss; T_10_: Temperature of 10% of weight loss; T_max_: Temperature of maximum rate of weight loss. The subscripts I and II refer to the first and second degradation stages. FD: Functionalization degree obtained from TGA thermograms.
